# Effects of Extracorporeal Shock Wave Therapy on Tendon Integrity, Biomechanical Strength, Matrix Remodeling, Inflammation, Angiogenesis, and Tenogenic Differentiation in Rotator Cuff Injury

**DOI:** 10.1002/kjm2.70260

**Published:** 2026-07-14

**Authors:** Hua Wang, Fei Wu, Jia‐Qing Miao

**Affiliations:** ^1^ Department of Orthopedics Putuo People's Hospital, School of Medicine, Tongji University Shanghai China

**Keywords:** angiogenesis, extracorporeal shock wave therapy, rotator cuff injury, tendon‐bone healing, tenogenic differentiation

## Abstract

Extracorporeal shock wave therapy (ESWT) improves tendon‐bone healing after rotator cuff injury (RCI), but its underlying cellular mechanisms remain unknown. This study aimed to explore the therapeutic effects of ESWT on RCI, with a particular focus on endothelial cells and tenocytes. The RCI model was established in rabbits by rotator cuff tear surgery, followed by ESWT intervention. Histological evaluation, blood perfusion analysis, biomechanical testing, and assessment of inflammation and extracellular matrix (ECM) degradation were performed in RCI‐treated rabbits at week (W) 2, W4, and W8. For the in vitro experiments, human umbilical vein endothelial cells (HUVECs) and human tenocytes were treated with ESWT. ESWT reduced Bonar scores, increased the fibrocartilage area and collagen‐positive area, and enhanced blood perfusion and biomechanical strength at most time points in RCI rabbits (all *p* < 0.05). Moreover, ESWT reduced inflammation and ECM degradation, as reflected by reduced tumor necrosis factor (TNF)‐α, interleukin (IL)‐1β, and IL‐6 and decreased matrix metalloproteinase (MMP)‐1 and MMP‐9 at most time points in RCI rabbits (all *p* < 0.05). In HUVECs, ESWT increased proliferation, invasion, branch points, MMP‐2, MMP‐9, vascular endothelial growth factor A (VEGFA), and fibroblast growth factor 2 (FGF2) (all *p* < 0.05). In tenocytes, ESWT increased proliferation, collagen I, thrombospondin‐4 (TSP4), VEGFA, FGF2, tenomodulin (TNMD), and scleraxis (SCX) (all *p* < 0.05). ESWT improves tendon integrity, blood perfusion, and biomechanical strength while reducing inflammation and ECM degradation after RCI, possibly via enhanced angiogenic activity in endothelial cells and maintenance of the tenogenic phenotype in tenocytes.

## 1 Introduction

Rotator cuff injury (RCI), one of the most common shoulder disorders, is characterized by progressive tendon degeneration and impaired tendon‐bone healing, leading to persistent pain and functional impairment [1]. Current treatment strategies for RCI include conservative management and surgery [2, 3, 4]. However, conservative treatment often provides only temporary symptom relief without halting disease progression [5]. Although surgical repair can restore tendon continuity, it is frequently complicated by postoperative pain, stiffness, and a high retear rate [6, 7, 8, 9]. Therefore, exploring potential therapeutic approaches is essential for improving the management of patients with RCI.

Extracorporeal shock wave therapy (ESWT) is a noninvasive physical modality that has been widely used in musculoskeletal disorders and has demonstrated beneficial effects on pain relief, osteogenesis, angiogenesis, and tissue repair [10, 11, 12]. Clinical studies and meta‐analyses have reported favorable efficacy and safety of ESWT in the treatment of RCI [13, 14, 15, 16]. For instance, Wang et al. reported that compared with conventional physical therapy, radial ESWT improved pain, shoulder function, and range of motion in patients with RCI [13]. Additionally, a recent meta‐analysis involving 1093 patients demonstrated that ESWT alleviated pain and improved functional outcomes in patients with RCI [14]. Moreover, several in vivo studies have suggested that ESWT may facilitate tendon‐bone healing, potentially through the modulation of extracellular matrix (ECM) organization, gene expression, and angiogenesis [17, 18, 19].

In addition to RCI models, the biological effects of ESWT have been investigated in other tendon injury models, such as Achilles tendon disorders [20, 21, 22]. For example, a previous study reported that ESWT promoted Achilles tendon healing in rats, potentially through the regulation of transforming growth factor‐beta 1 (TGF‐β1) and insulin‐like growth factor I (IGF‐I) [20]. In a rabbit Achilles tendon‐bone junction model, ESWT induced neovascularization and increased the expression of angiogenesis‐related markers, suggesting enhanced tissue regeneration at the tendon‐bone interface [22]. Moreover, an in vitro study revealed that ESWT reduced inflammation in tenocytes, revealing a potential mechanism of ESWT in tendinopathy [23]. Collectively, these studies suggest that ESWT has beneficial effects across different tendon injury models. However, RCI is characterized by a unique tendon‐bone healing environment and complex cellular interactions, and the cellular mechanisms underlying ESWT‐mediated repair in RCI remain incompletely understood. Therefore, further investigations focusing on key cell populations involved in RCI healing are warranted.

Therefore, the present study aimed to evaluate the effects of ESWT on tendon‐bone healing in RCI and to further investigate its potential cellular mechanisms, with a particular focus on endothelial cells and tenocytes.

## 2 Materials and Methods

### 2.1 Animals and RCI Model Construction

**FIGURE 1:**
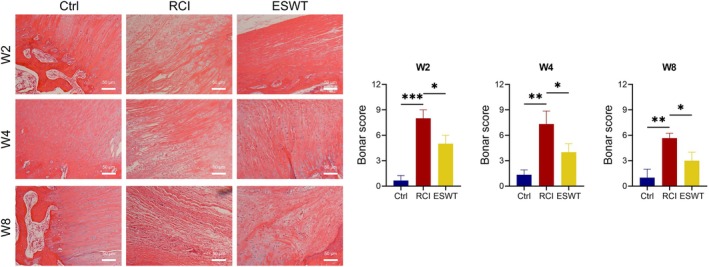
Effects of ESWT on tendon degeneration in RCI rabbits. *p < 0.05; **p < 0.01; ***p < 0.001.

Fifty‐four New Zealand white rabbits (2.0–2.2 kg, male) were purchased from Hunan Taiping Biotechnology Co. Ltd. (Hunan, China). Following a 7‐day acclimation period, the RCI model was established in the rabbits by rotator cuff tear surgery according to previously described methods [24]. Briefly, the rabbits were anesthetized, and a longitudinal skin incision was made on the left shoulder. The deltoid muscle was bluntly separated to expose the tendon. A 0.5 × 0.5 cm segment of tendon tissue was excised, creating a full‐thickness rotator cuff defect. A black suture was placed at the inferior margin of the rotator cuff for anatomical orientation. The establishment of the RCI model is shown in Figure 1. All experimental procedures were approved by the Ethics Committee and carried out in compliance with applicable guidelines.

### 2.2 ESWT Intervention In Vivo

The rabbits were randomly divided into three groups (*n* = 18 per group): the control group (Ctrl), RCI group, and ESWT group. In the Ctrl group, the tendon was exposed without tissue removal. In the RCI group and ESWT groups, an RCI model was established. Furthermore, rabbits in the ESWT group received ESWT once weekly (0.1 mJ/mm^2^, 1500 impulses, 3 Hz) using a focused shock wave therapy apparatus (Xiangyu Medical, China) [25]. The focal length was adjusted to target the supraspinatus tendon region without setting a fixed focal distance.

At 2, 4, and 8 weeks (W2, W4, and W8) after surgery, six rabbits per group were randomly selected at each time point and euthanized via intravenous overdose of pentobarbital sodium. Blood perfusion measurements were performed before euthanasia. The rabbits in the W2, W4, and W8 groups received ESWT for 2, 4, and 8 weeks, respectively. Synovial fluid was collected from the shoulder joint cavity on the surgical side for use in an enzyme‐linked immunosorbent assay (ELISA). The entire supraspinatus–humerus complex of the surgical side, including the humeral head, tendon‐bone insertion site, and supraspinatus muscle, was harvested for pathological staining and biomechanical testing.

### 2.3 Hematoxylin and Eosin (H&E), Safranin O/Fast Green, and Sirius Red Staining

The entire supraspinatus–humerus complex was fixed in 10% formalin, followed by decalcification with an EDTA Decalcification Kit (Servicebio, China). The samples were then dehydrated, embedded, and sectioned. For H&E staining, sections were exposed to hematoxylin reagent and eosin reagent (Servicebio, China), and the Bonar score was assessed using methods previously described [26]. Fibrocartilage formation at the tendon‐bone interface was evaluated via a Safranin O/Fast Green Staining Kit (Servicebio, China). The metachromatic fibrocartilage region was identified microscopically, and the area of fibrocartilage was quantified using ImageJ (NIH, USA) [27]. Sirius Red staining was carried out using the Modified Sirius Red Staining Kit (Servicebio, China) to evaluate collagen deposition and organization, and the collagen‐positive area was quantified using ImageJ [27].

### 2.4 Blood Perfusion Measurement

Local blood perfusion at the supraspinatus tendon on the surgical side was measured using a MoorLD12 laser Doppler imaging system (Moor Instruments, UK). In brief, the rabbits were anesthetized and positioned in a standardized prone posture. The probe was maintained at a fixed distance from the supraspinatus tendon to ensure consistency. Perfusion values were recorded.

### 2.5 Biomechanical Testing

Biomechanical testing was used to assess the structural strength of the supraspinatus muscle. Briefly, the supraspinatus muscle belly was carefully separated from the surrounding tissue while preserving its structural integrity. Each sample was mounted onto a uniaxial tensile testing machine (Instron, USA). The samples were stretched (0.1 mm/s), and the maximum load (N) was recorded.

### 2.6 Elisa

Synovial fluid was centrifuged to remove debris. The concentrations of tumor necrosis factor (TNF)‐α, interleukin (IL)‐1β, IL‐6, matrix metalloproteinase (MMP)‐1, and MMP‐9 were quantified via ELISA kits (mlbio, China) following the kit protocol.

### 2.7 ESWT Intervention In Vitro

Commercially sourced human umbilical vein endothelial cells (HUVECs; Procell, China) and human tenocytes (Cellverse, China) were expanded in high‐glucose complete medium (DMEM; Servicebio, China) at 37°C and 5% CO_2_. HUVECs and tenocytes were subjected to ESWT. Briefly, cells were seeded and allowed to reach 80% confluence. The culture plates were sealed with sterile parafilm to prevent contamination and positioned in direct contact with the applicator head using sterile ultrasound coupling gel (HYNAUT, China) to ensure efficient energy transmission. Shock waves were delivered at 0.14 mJ/mm^2^ for 1000 impulses [28]. Cells without shock wave exposure served as the Ctrl group. Following ESWT, the cells were subjected to further assays.

### 2.8 Cell Proliferation Assay

After ESWT intervention, HUVECs and tenocytes were plated (3 × 10^3^ cells/well). At 0, 24, 48, and 72 h, a Cell Counting Kit‐8 solution (Dojindo, China) was used for 2 h. The optical density (OD) values (450 nm) were subsequently read by a microplate reader (Huadong‐Electronics, China).

### 2.9 Transwell Invasion Assay

After ESWT intervention, the HUVECs were resuspended in serum‐free medium (5 × 10^4^ cells) and added to Matrigel‐precoated Transwell chambers (Corning, USA), after which complete medium was added to the lower chambers. After 24 h of incubation, the cells that invaded through the membrane were stained and counted.

### 2.10 Tube Formation Assay

After ESWT intervention, HUVECs (1 × 10^4^ cells/well) were seeded onto Matrigel‐coated plates. After incubation for 6 h, tubular structures formed and were captured under a microscope. Angiogenesis was quantified by calculating the number of branch points via ImageJ.

### 2.11 Western Blot Analysis

Protein from HUVECs and tenocytes was isolated via a RIPA Kit (Servicebio, China). The proteins were electrophoresed and transferred to membranes (Solarbio, China). The membranes were blocked and then incubated with primary antibodies against MMP‐2 (1:2000; Proteintech, China), MMP‐9 (1:2000; Proteintech, China), vascular endothelial growth factor A (VEGFA) (1:2500; Proteintech, China), fibroblast growth factor 2 (FGF2) (1:2000; ABclonal, China), collagen I (1:800; Affinity, China), collagen III (1:1000; Affinity, China), thrombospondin‐4 (TSP4) (1:500; Absin, China), tenomodulin (TNMD) (1:2500; ABclonal, China), scleraxis (SCX) (1:1000; Affinity, China), and GAPDH (1:2000; Affinity, China). After being exposed to secondary antibody (1:3000; Servicebio, China) for 1 h, the blots were visualized via enhanced chemiluminescence (Thermo Fisher, China) and quantified by ImageJ.

### 2.12 Statistical Analysis

The data are expressed as the mean ± standard deviation (SD). Statistical analyses were carried out via GraphPad Prism software (V10; Dotmatics, USA). Comparisons between two groups were analyzed using Student's *t*‐test, and comparisons among multiple groups were conducted using one‐way analysis of variance (ANOVA) followed by Tukey's post hoc test. A value of *p* < 0.05 was considered to indicate statistical significance.

## 3 Results

### 3.1 ESWT Alleviated Tendon Degeneration in RCI Rabbits

H&E staining revealed that in the Ctrl group, the tissue architecture was intact, with well‐organized collagen fibers, normal cell morphology, uniform nuclear staining, and no evident inflammatory cell infiltration. The tendon‐bone interface appeared continuous and well defined. In contrast, the RCI group exhibited marked structural disruption, including disorganized collagen fibers, focal fiber rupture with increased interfibrillar spacing, and prominent inflammatory cell infiltration. The continuity of the tendon‐bone interface was impaired, indicating tendon degeneration and delayed healing. However, these histological alterations improved in the ESWT group compared with the RCI group; in detail, collagen fibers appeared more regularly aligned, inflammatory cell infiltration was reduced, and partial restoration of the tendon‐bone interface was observed. Quantitative analysis revealed that Bonar scores at W2, W4, and W8 were higher in the RCI group than in the Ctrl group (all *p* < 0.01) but lower in the ESWT group than in the RCI group (all *p* < 0.05) (Figure 1).

### 3.2 ESWT Improved Tendon Fibrocartilage Formation and Collagen Deposition in RCI Rabbits

**FIGURE 2:**
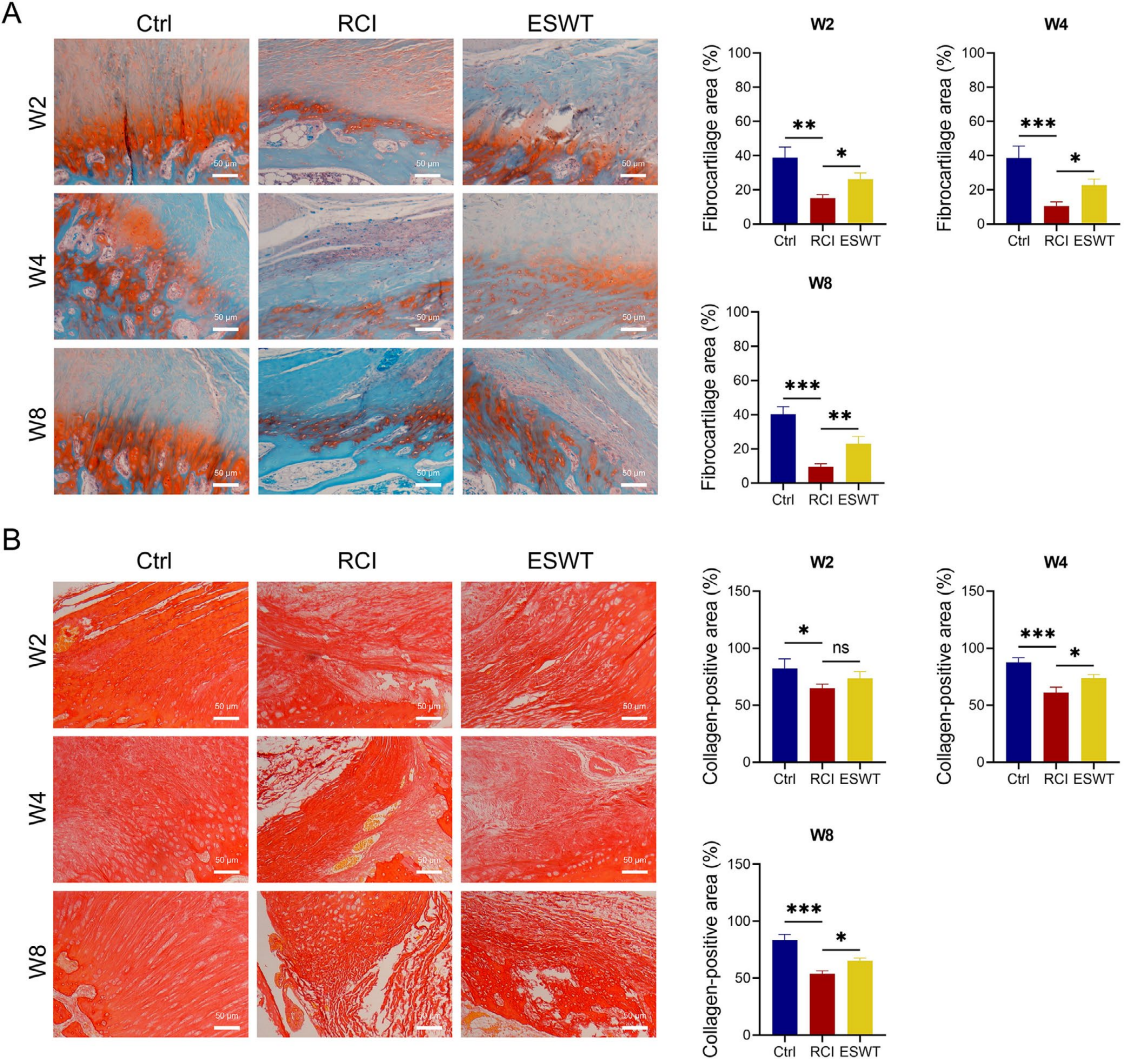
Effects of ESWT on tendon fibrocartilage formation and collagen deposition in RCI rabbits. Safranin O/Fast Green staining and quantitative analysis of the fibrocartilage area at W2, W4, and W8 among the Ctrl, RCI, and ESWT groups (A). Sirius Red staining and quantitative analysis of the collagen‐positive area at W2, W4, and W8 among the Ctrl, RCI, and ESWT groups (B). *p < 0.05; **p < 0.01; ***p < 0.001; ns, *p* > 0.05.

Safranin O/Fast Green staining revealed intense and uniform Safranin O staining in the Ctrl group, with a well‐defined and continuous interface. The fibrocartilage layer was intact, with regularly arranged cells, indicating abundant proteoglycan content and normal tissue architecture. The RCI group showed markedly reduced Safranin O staining, a decreased red‐stained area, and a disrupted tendon‐bone interface. The fibrocartilage layer appeared thinned or discontinuous, accompanied by a disorganized collagen structure and degenerative changes. Compared with those in the RCI group, the Safranin O staining intensity and red‐stained area in the ESWT group increased, and the interface structure became more organized with improved continuity. Quantitative analysis confirmed that the fibrocartilage area at W2, W4, and W8 was decreased in the RCI group compared with that in the Ctrl group (all *p* < 0.01), whereas it was increased in the ESWT group compared with that in the RCI group (all *p* < 0.05) (Figure 2A).

With respect to collagen deposition, Sirius Red staining revealed that in the Ctrl group, the collagen fibers were densely packed, well organized, and continuously distributed. In the RCI group, collagen fibers were markedly disrupted, with a disorganized arrangement and increased interfibrillar spacing, indicating progressive degeneration at the tendon‐bone interface. Collagen organization was improved in the ESWT group compared with the RCI group, as evidenced by more aligned fiber orientation and reduced structural disruption. Quantitative analysis revealed that the collagen‐positive area was reduced in the RCI group compared with that in the Ctrl group at W2, W4, and W8 (all *p* < 0.05) but was increased in the ESWT group compared with that in the RCI group at W4 and W8 (both *p* < 0.05) (Figure 2B).

### 3.3 ESWT Enhanced Local Blood Perfusion in RCI Rabbits

**FIGURE 3:**
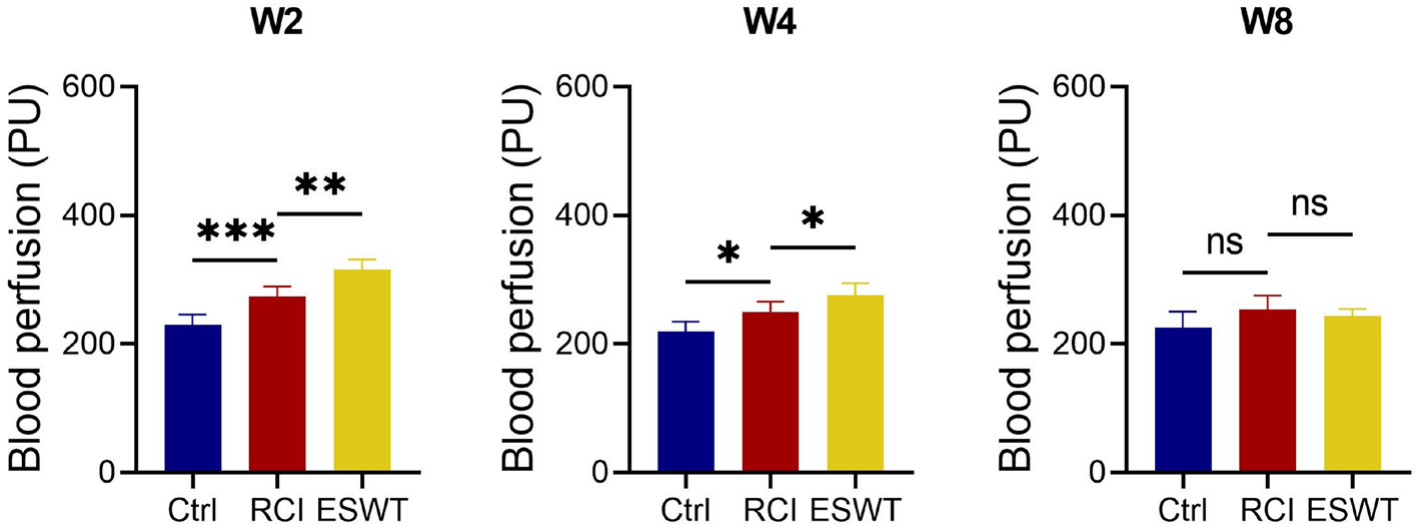
Effects of ESWT on blood perfusion in RCI rabbits. *p < 0.05; **p < 0.01; ***p < 0.001; ns, *p* > 0.05.

At W2 and W4, blood perfusion was greater in the RCI group than in the Ctrl group (both *p* < 0.05), whereas it was further greater in the ESWT group than in the RCI group (both *p* < 0.05). However, blood perfusion at W8 did not differ among the groups (*p* > 0.05) (Figure 3).

### 3.4 ESWT Improved Tendon Biomechanical Strength in RCI Rabbits

**FIGURE 4:**
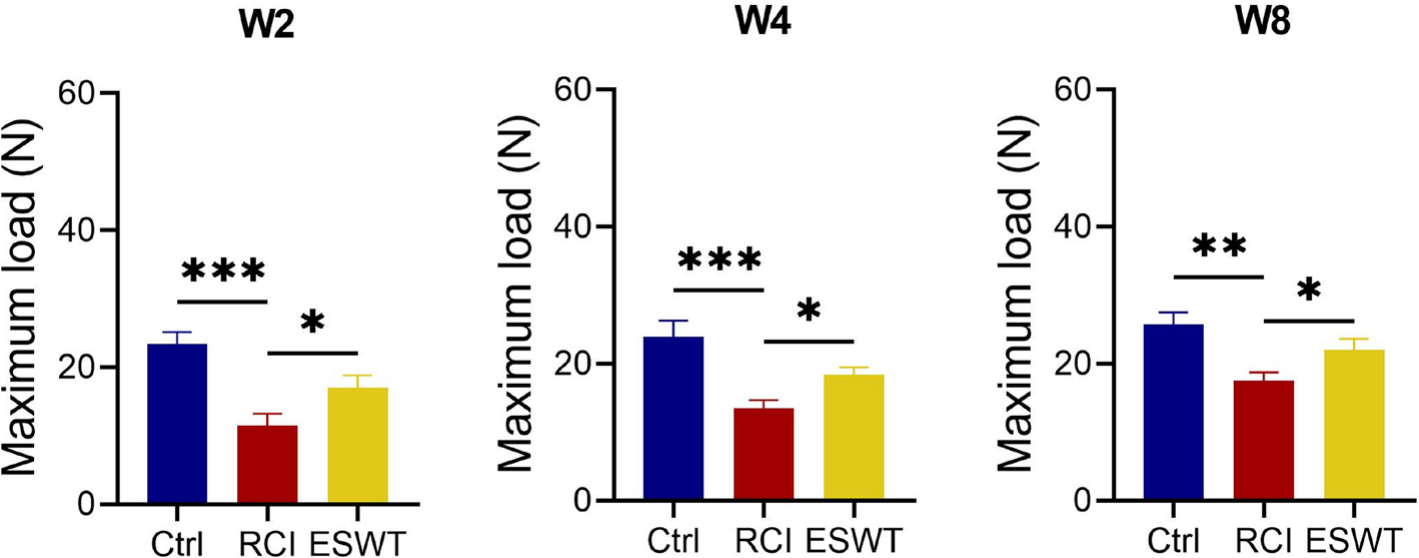
Effects of ESWT on tendon biomechanical strength in RCI rabbits. *p < 0.05; **p < 0.01; ***p < 0.001.

The maximum load at W2, W4, and W8 decreased in the RCI group compared with that in the Ctrl group (all *p* < 0.01), but it increased in the ESWT group compared with that in the RCI group at all time points (all *p* < 0.05) (Figure 4).

### 3.5 ESWT Attenuated Inflammation and ECM Degradation in RCI Rabbits

**FIGURE 5:**
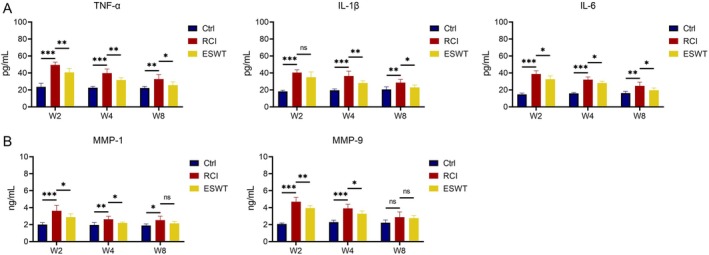
Effects of ESWT on inflammation and ECM degradation in RCI rabbits. Comparisons of TNF‐α, IL‐1β, and IL‐6 at W2, W4, and W8 among the Ctrl, RCI, and ESWT groups (A). Comparisons of MMP‐1 and MMP‐9 at W2, W4, and W8 among the Ctrl, RCI, and ESWT groups (B). *p < 0.05; **p < 0.01; ***p < 0.001; ns, *p* > 0.05.

Regarding inflammation, TNF‐α, IL‐1β, and IL‐6 at W2, W4, and W8 were increased in the RCI group versus the Ctrl group (all *p* < 0.01). However, TNF‐α and IL‐6 at W2, W4, and W8, as well as IL‐1β at W4 and W8, were decreased in the ESWT group versus the RCI group (all *p* < 0.05) (Figure 5A).

For ECM degradation, MMP‐1 at all time points and MMP‐9 at W2 and W4 were increased in the RCI group versus the Ctrl group (all *p* < 0.05). However, MMP‐1 and MMP‐9 at W2 and W4 were decreased in the ESWT group versus the RCI group (all *p* < 0.05) (Figure 5B).

### 3.6 ESWT Facilitated Proliferation, Invasion, and Angiogenesis in HUVECs

**FIGURE 6:**
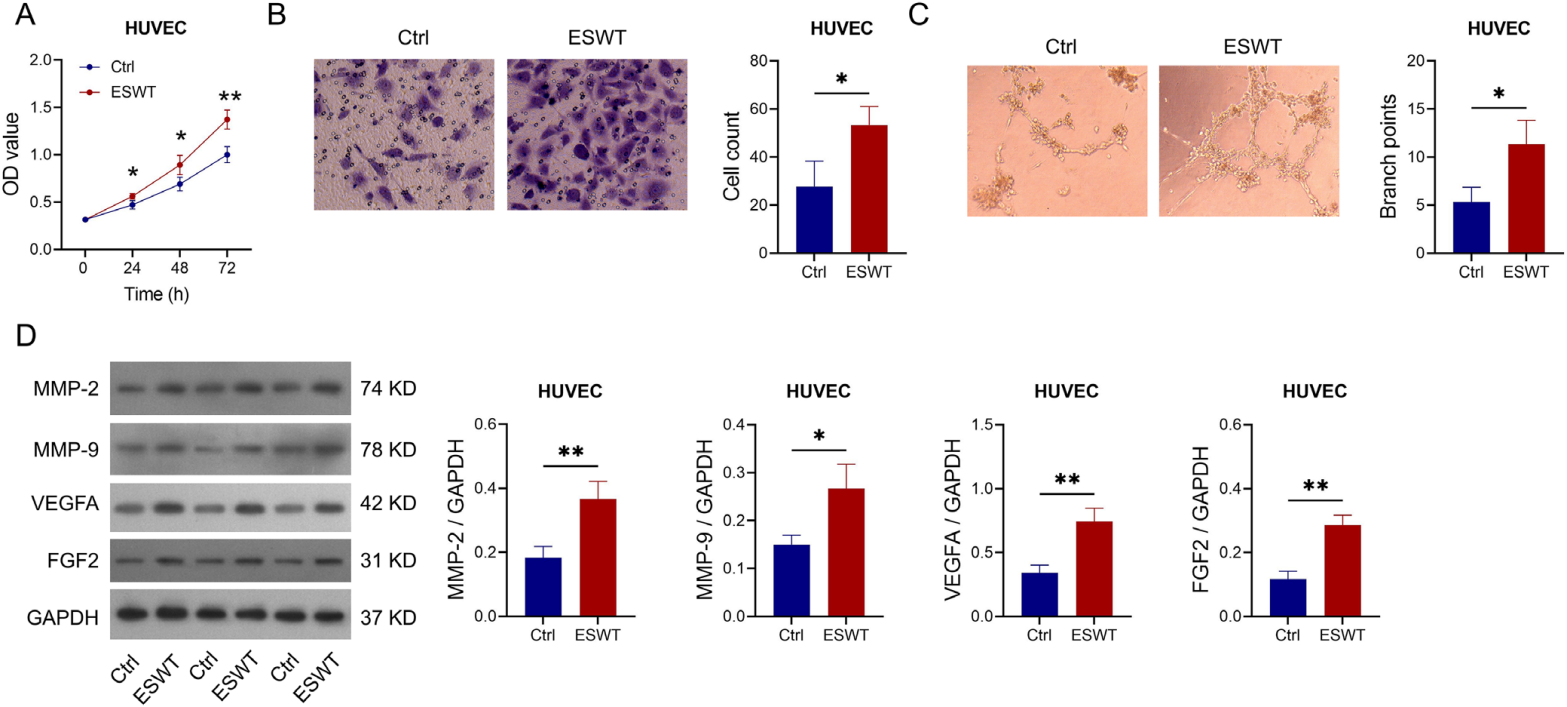
Effects of ESWT on proliferation, invasion, and angiogenesis in HUVECs. Comparisons of proliferation (A), invasion (B), branch points (C), and MMP‐2, MMP‐9, VEGFA, and FGF2 (D) between the Ctrl and ESWT groups. *p < 0.05; **p < 0.01.

HUVEC proliferation at 24, 48, and 72 h was greater in the ESWT group than in the Ctrl group (all *p* < 0.05) (Figure 6A). In addition, HUVEC invasion was improved in the ESWT group versus the Ctrl group (*p* < 0.05) (Figure 6B). Branch points were increased in the ESWT group versus the Ctrl group (*p* < 0.05) (Figure 6C). Regarding angiogenesis‐related markers, MMP‐2, MMP‐9, VEGFA, and FGF2 were all increased in the ESWT group versus the Ctrl group (all *p* < 0.05) (Figure 6D).

### 3.7 ESWT Promoted Proliferation, Matrix Remodeling, and Tenogenic Marker Expression in Tenocytes

**FIGURE 7:**
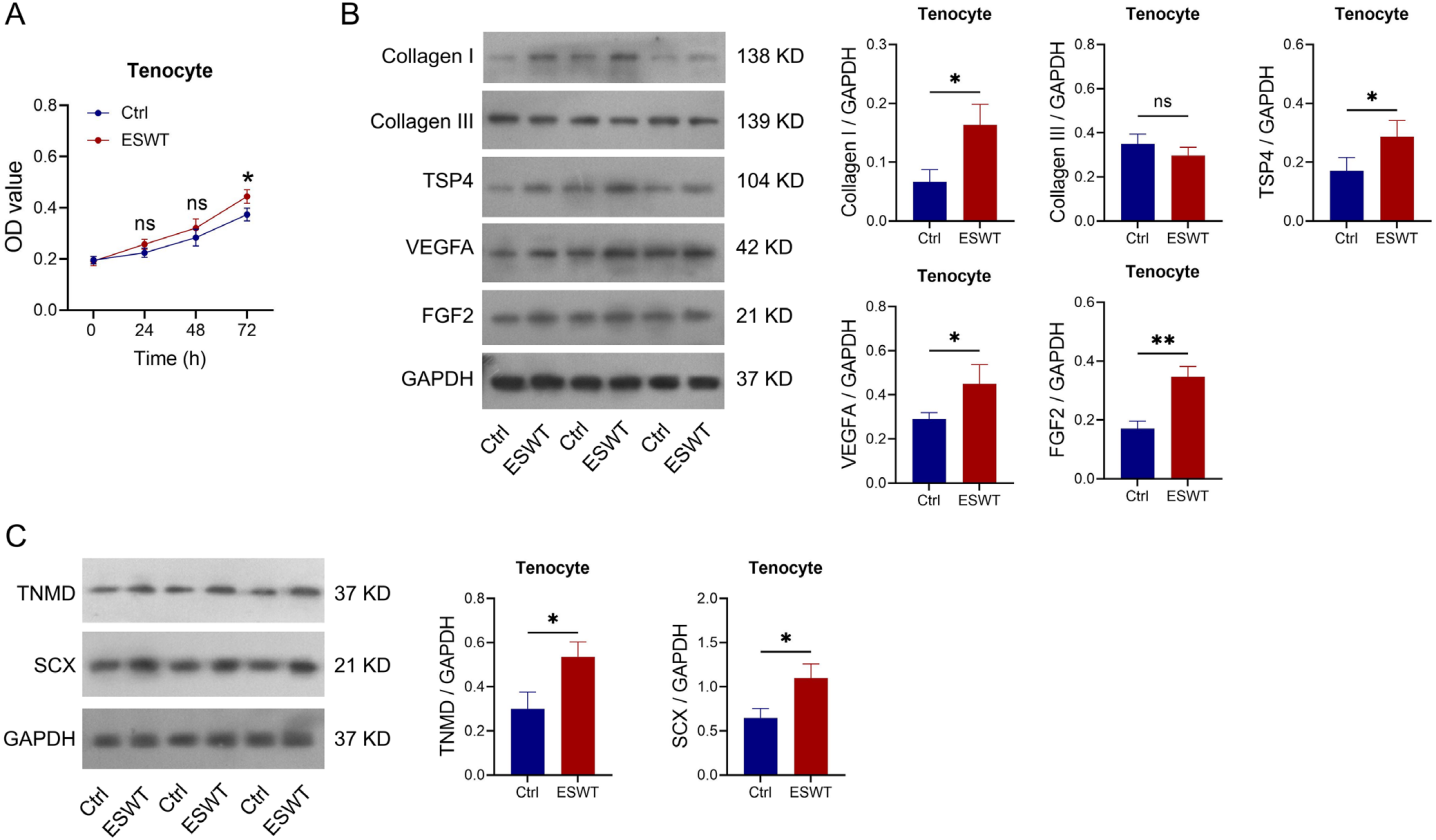
Effects of ESWT on proliferation, matrix remodeling, and tenogenic marker expression in tenocytes. Comparisons of proliferation (A), collagen I, collagen III, TSP4, VEGFA, and FGF2 (B), and TNMD and SCX (C) between the Ctrl and ESWT groups. *p < 0.05; **p < 0.01; ns, *p* > 0.05.

Compared with that in the Ctrl group, tenocyte proliferation at 72 h was increased in the ESWT group (*p* < 0.05) (Figure 7A). Collagen I, TSP4, VEGFA, and FGF2 were increased in the ESWT group compared with the Ctrl group (all *p* < 0.05) (Figure 7B). With respect to tenogenic differentiation‐related markers, TNMD and SCX were elevated in the ESWT group compared with those in the Ctrl group (both *p* < 0.05) (Figure 7C).

## 4 Discussion

RCI disrupts the structural integrity of the tendon‐bone interface, characterized by disorganized collagen fibers, enhanced ECM degradation, and reduced mechanical strength [29]. Previous studies have demonstrated that ESWT can promote structural repair and improve the biomechanical properties of injured tendons [17, 30]. For instance, Feichtinger et al. [30] reported that ESWT significantly increased the maximum failure load in chronic rotator cuff tear rats, while Kamiyama et al. [17] found that ESWT improved collagen fiber organization. Consistent with these findings, we found that ESWT reduced Bonar scores, increased fibrocartilage formation and collagen deposition, and improved the maximum load of the supraspinatus tendon in RCI rabbits. Additionally, we observed that ESWT enhanced local blood perfusion in RCI rabbits, which might be related to the ability of ESWT to promote neovascularization [31, 32]. Notably, no difference in blood perfusion at W8 was observed between the ESWT and RCI groups. This might be attributed to vascular maturation and remodeling during the later stage of healing, which could attenuate the effects of ESWT on blood perfusion. With respect to inflammation, ESWT reduced TNF‐α, IL‐1β, and IL‐6 in RCI rabbits. On the basis of previous evidence, we speculated that the anti‐inflammatory effects of ESWT may be regulated through the modulation of nitric oxide production and the regulation of macrophage activity [33]. Moreover, ESWT reduced f MMP‐1 and MMP‐9 in RCI rabbits. These findings suggested that ESWT could attenuate ECM degradation and improve tendon‐bone structural integrity after RCI. Tear size is an important indicator for evaluating the extent of tendon healing following RCI. However, tear size was not measured prior to biomechanical testing in the present study because the original defect area was covered by newly formed repair tissue at the time of harvest, making accurate identification of the initial tear margins difficult and preventing reliable measurement of the residual defect size.

Endothelial cell proliferation and invasion are essential steps in angiogenesis and are critical for microvascular reconstruction during tissue repair [34]. According to previous studies, ESWT could enhance endothelial cell proliferation and invasion [35, 36]. In accordance with these previous studies, we also observed that ESWT increased HUVEC proliferation and invasion. A potential reason might be that ESWT could upregulate VEGF expression and activate downstream pathways, such as extracellular signal‐regulated kinase (ERK)/mitogen‐activated protein kinase (MAPK) and phosphatidylinositol 3‐kinase (PI3K)/protein kinase B (AKT), to enhance endothelial cell proliferation and invasion [35, 36]. In addition, ESWT promoted angiogenic capacity, as evidenced by increased branch points and upregulation of angiogenesis‐related proteins, including MMP‐2, MMP‐9, VEGFA, and FGF2. These effects might be attributed to VEGF upregulation and activation of some downstream pathways, including ERK and PI3K/AKT pathways [35, 37]. Notably, the effects of ESWT on MMP‐1 and MMP‐9 were inconsistent between our in vivo and in vitro experiments, which might be attributed to differences in cellular context and healing stage. The in vitro model reflects direct proangiogenic effects of ESWT on endothelial cells, whereas the in vivo model reflects a later, multicellular environment in which reduced inflammation by ESWT might lead to reduced MMP expression.

In addition to endothelial cells, tenocytes play a key role in tendon‐bone healing after RCI [38]. The effects of ESWT on tenocytes have been reported previously [28, 39]. For instance, Vetrano et al. [28] reported that ESWT enhanced tenocyte proliferation and the synthesis of collagen, particularly Type I collagen, in primary cultured human tenocytes. Chao et al. [39] demonstrated that ESWT stimulated tenocyte proliferation and increased the expression of collagen I, collagen III, and transforming growth factor‐β1. In line with these previous studies, ESWT promoted tenocyte proliferation in the present study. Additionally, matrix‐related proteins (Collagen I and TSP4) and proangiogenic factors (VEGFA and FGF2) increased in response to ESWT, indicating enhanced matrix remodeling capacity. Moreover, we discovered that ESWT upregulated the tenogenic markers TNMD and SCX in tenocytes. However, Leone et al. reported that SCX was reduced by ESWT in tenocytes [40]. This discrepancy might be related to differences in cell source, as Leone et al. used tenocytes derived from ruptured tendons, whereas we used tenocytes without any prior injury, potentially leading to a different response to ESWT. In this study, in vitro experiments were performed using commercially available human tenocytes rather than primary rabbit‐derived tenocytes. This approach was adopted because the isolation and culture of primary rabbit tenocytes were technically challenging, with limited cell yield, considerable inter‐animal variability, and potential phenotypic drift during expansion, which might compromise experimental consistency and reproducibility. Nevertheless, the use of primary rabbit tenocytes would provide a species‐matched in vitro model. Future studies incorporating primary rabbit tenocytes might help further validate the biological relevance of our findings.

In this study, the ESWT parameters used in the in vivo and in vitro experiments were not identical because the two experimental models had different biological characteristics and required separate parameter optimization. In the present study, in vivo and in vitro ESWT settings were selected with reference to previous studies investigating the effects of ESWT in animal models [25] and cultured cells [28], as well as our preliminary experiments. Therefore, the ESWT parameters were optimized independently for the in vivo and in vitro experiments rather than being unified across the two models.

Overall, the present study demonstrated that ESWT alleviated tendon degeneration, enhanced blood perfusion and biomechanical strength, and reduced inflammation and ECM degradation in RCI rabbits. In addition, ESWT promoted angiogenic activity in endothelial cells and supported matrix remodeling and maintenance of the tenogenic phenotype in tenocytes. Collectively, these findings suggest that ESWT may facilitate RCI repair through the regulation of endothelial cell and tenocyte functions.

## 5 Conclusion

In conclusion, ESWT promotes tendon integrity, blood perfusion, and biomechanical strength while reducing inflammation and ECM degradation after RCI, potentially through enhancing angiogenic activity in endothelial cells and maintaining the tenogenic phenotype in tenocytes.

## Funding

This study was supported by the Science and Technology Innovation Project of the Health and Wellness System in Putuo District, Shanghai (No. ptkwws202205) and The New Round (2023–2026) Clinical Medicine Discipline Construction Plan of the Health and Wellness System in Putuo District, Shanghai (No. 2024tszb06).

## Ethics Statement

All experimental procedures were approved by the Science and Technology Ethics Committee of Tongji University (Approval No. TJBJ00325301) and carried out in compliance with applicable guidelines.

## Conflicts of Interest

The authors declare no conflicts of interest.

## Supporting information


**Figure 1** Schematic diagram of RCI model establishment.

## Data Availability

The data that support the findings of this study are available from the corresponding author upon reasonable request.

## References

[1] A. Bedi , J. Bishop , J. Keener , et al., “Rotator Cuff Tears,” Nature Reviews Disease Primers 10, no. 1 (2024): 8. 10.1038/s41572-024-00492-3 38332156

[2] S. Lafrance , M. Charron , J. S. Roy , et al., “Diagnosing, Managing, and Supporting Return to Work of Adults With Rotator Cuff Disorders: A Clinical Practice Guideline,” Journal of Orthopaedic and Sports Physical Therapy 52, no. 10 (2022): 647–664. 35881707 10.2519/jospt.2022.11306

[3] V. Lowry , P. Lavigne , D. Zidarov , E. Matifat , A. A. Cormier , and F. Desmeules , “A Systematic Review of Clinical Practice Guidelines on the Diagnosis and Management of Various Shoulder Disorders,” Archives of Physical Medicine and Rehabilitation 105, no. 2 (2024): 411–426. 37832814 10.1016/j.apmr.2023.09.022

[4] R. N. Dickinson and J. E. Kuhn , “Nonoperative Treatment of Rotator Cuff Tears,” Physical Medicine and Rehabilitation Clinics of North America 34, no. 2 (2023): 335–355. 37003656 10.1016/j.pmr.2022.12.002

[5] U. G. Longo , A. Lalli , G. Medina , and N. Maffulli , “Conservative Management of Partial Thickness Rotator Cuff Tears: A Systematic Review,” Sports Medicine and Arthroscopy Review 31, no. 3 (2023): 80–87. 37976129 10.1097/JSA.0000000000000372

[6] M. R. Mancini , J. L. Horinek , C. J. Phillips , and P. J. Denard , “Arthroscopic Rotator Cuff Repair: A Review of Surgical Techniques and Outcomes,” Clinics in Sports Medicine 42, no. 1 (2023): 81–94. 36375872 10.1016/j.csm.2022.08.004

[7] C. A. Uquillas , B. M. Capogna , W. H. Rossy , S. A. Mahure , and A. S. Rokito , “Postoperative Pain Control After Arthroscopic Rotator Cuff Repair,” Journal of Shoulder and Elbow Surgery 25, no. 7 (2016): 1204–1213. 27079219 10.1016/j.jse.2016.01.026

[8] M. Salas , B. Zaldivar , G. Fierro , J. C. Gonzalez , and J. R. Lievano , “Incidence and Risk Factors for Shoulder Stiffness After Open and Arthroscopic Rotator Cuff Repair,” Archives of Orthopaedic and Trauma Surgery 144, no. 5 (2024): 2047–2055. 38630250 10.1007/s00402-024-05323-4

[9] K. Yamaura , I. Fujibayashi , T. Kurosawa , et al., “Timing of Retears After Arthroscopic Rotator Cuff Repair and Associated Factors: A Retrospective Analysis,” Journal of Shoulder and Elbow Surgery 32, no. 9 (2023): 1929–1936. 36842463 10.1016/j.jse.2023.01.026

[10] H. De la Corte‐Rodríguez , J. M. Román‐Belmonte , B. A. Rodríguez‐Damiani , A. Vázquez‐Sasot , and E. C. Rodríguez‐Merchán , “Extracorporeal Shock Wave Therapy for the Treatment of Musculoskeletal Pain: A Narrative Review,” Health 11, no. 21 (2023): 2830. 10.3390/healthcare11212830 PMC10648068 37957975

[11] Z. Qiu , J. Wang , Y. Zhang , X. Liu , C. Wei , and T. Ma , “Extracorporeal Shock Wave Therapy for Equine Musculoskeletal Disorders: From Biological Mechanisms to Clinical Applications,” Frontiers in Veterinary Science 12 (2025): 1719123. 41487475 10.3389/fvets.2025.1719123 PMC12757233

[12] R. Charles , L. Fang , R. Zhu , and J. Wang , “The Effectiveness of Shockwave Therapy on Patellar Tendinopathy, Achilles Tendinopathy, and Plantar Fasciitis: A Systematic Review and Meta‐Analysis,” Frontiers in Immunology 14 (2023): 1193835. 37662911 10.3389/fimmu.2023.1193835 PMC10468604

[13] Z. Wang , L. Tang , N. Wang , et al., “Radial Extracorporeal Shock Wave Therapy Versus Multimodal Physical Therapy in Non‐Traumatic (Degenerative) Rotator Cuff Tendinopathy With Partial Supraspinatus Tear: A Randomized Controlled Trial,” Journal of Clinical Medicine 15, no. 2 (2026): 471. 41598412 10.3390/jcm15020471 PMC12841779

[14] X. Xue , Q. Song , X. Yang , et al., “Effect of Extracorporeal Shockwave Therapy for Rotator Cuff Tendinopathy: A Systematic Review and Meta‐Analysis,” BMC Musculoskeletal Disorders 25, no. 1 (2024): 357. 38704572 10.1186/s12891-024-07445-7 PMC11069249

[15] H. Shao , S. Zhang , J. Chen , et al., “Radial Extracorporeal Shockwave Therapy Reduces Pain and Promotes Proximal Tendon Healing After Rotator Cuff Repair: Randomized Clinical Trial,” Annals of Physical and Rehabilitation Medicine 66, no. 4 (2023): 101730. 37027927 10.1016/j.rehab.2023.101730

[16] D. H. Kamonseki , G. M. da Rocha , V. Ferreira , J. M. Ocarino , and L. S. Pogetti , “Extracorporeal Shockwave Therapy for the Treatment of Noncalcific Rotator Cuff Tendinopathy: A Systematic Review and Meta‐Analysis,” American Journal of Physical Medicine & Rehabilitation 103, no. 6 (2024): 471–479. 37903597 10.1097/PHM.0000000000002361

[17] M. Kamiyama , H. Shitara , T. Ichinose , et al., “Histological and Genetic Changes Induced by Extracorporeal Shockwave Therapy After Rotator Cuff Repair in a Rat Model With Tears,” Scientific Reports 16, no. 1 (2026): 5046. 41526482 10.1038/s41598-026-35072-w PMC12876060

[18] U. Demirtaş , E. M. Demirtaş , M. Özcan , and F. Puyan , “Comparative Effects of Bone Marrow Stimulation and Shockwave Therapy on Rotator Cuff Healing in Chronic Massive Rotator Cuff Tears: A Rat Model Study,” Journal of Experimental Orthopaedics 13, no. 1 (2026): e70622. 41531477 10.1002/jeo2.70622 PMC12793047

[19] X. Feichtinger , P. Heimel , S. Tangl , et al., “Improved Biomechanics in Experimental Chronic Rotator Cuff Repair After Shockwaves Is Not Reflected by Bone Microarchitecture,” PLoS One 17, no. 1 (2022): e0262294. 34986173 10.1371/journal.pone.0262294 PMC8730430

[20] Y. J. Chen , C. J. Wang , K. D. Yang , et al., “Extracorporeal Shock Waves Promote Healing of Collagenase‐Induced Achilles Tendinitis and Increase TGF‐beta1 and IGF‐I Expression,” Journal of Orthopaedic Research: Official Publication of the Orthopaedic Research Society 22, no. 4 (2004): 854–861. 15183445 10.1016/j.orthres.2003.10.013

[21] S. D. Yoo , S. Choi , G. J. Lee , et al., “Effects of Extracorporeal Shockwave Therapy on Nanostructural and Biomechanical Responses in the Collagenase‐Induced Achilles Tendinitis Animal Model,” Lasers in Medical Science 27, no. 6 (2012): 1195–1204. 22274874 10.1007/s10103-011-1049-0

[22] C. J. Wang , F. S. Wang , K. D. Yang , et al., “Shock Wave Therapy Induces Neovascularization at the Tendon‐Bone Junction. A Study in Rabbits,” Journal of Orthopaedic Research: Official Publication of the Orthopaedic Research Society 21, no. 6 (2003): 984–989. 14554209 10.1016/S0736-0266(03)00104-9

[23] R. Ge , B. Ruan , S. Chen , S. Bai , Q. Gao , and A. Dong , “Radial Extracorporeal Shock Wave Therapy Alleviates Acute Inflammation of Human Primary Tenocytes Through the Integrin‐FAK‐p38MAPK Pathway,” American Journal of Translational Research 15, no. 5 (2023): 3229–3239. 37303680 PMC10251031

[24] A. Inui , T. Kokubu , Y. Mifune , et al., “Regeneration of Rotator Cuff Tear Using Electrospun Poly(d,l‐Lactide‐Co‐Glycolide) Scaffolds in a Rabbit Model,” Arthroscopy: The Journal of Arthroscopic & Related Surgery: Official Publication of the Arthroscopy Association of North America and the International Arthroscopy Association 28, no. 12 (2012): 1790–1799. 23058811 10.1016/j.arthro.2012.05.887

[25] Y. J. Lee , Y. S. Moon , D. R. Kwon , S. C. Cho , and E. H. Kim , “Polydeoxyribonucleotide and Shock Wave Therapy Sequence Efficacy in Regenerating Immobilized Rabbit Calf Muscles,” International Journal of Molecular Sciences 24, no. 16 (2023): 12820. 37629001 10.3390/ijms241612820 PMC10454565

[26] R. Zhang , H. Li , Y. Mu , et al., “Turmeric‐Derived Extracellular Vesicles Loaded Microneedle System Attenuates Rotator Cuff Degeneration by Orchestrating Energetic Metabolism,” Materials Today Bio 35 (2025): 102590. 10.1016/j.mtbio.2025.102590 PMC12718207 41431730

[27] C. Du , W. Chen , J. Fang , et al., “Comparison of 3 Different Surgical Techniques for Rotator Cuff Repair in a Rabbit Model: Direct Suture, Inlay Suture, and Polyether Ether Ketone (PEEK) Suture Anchor,” American Journal of Sports Medicine 52, no. 6 (2024): 1428–1438. 38619003 10.1177/03635465241240140

[28] M. Vetrano , F. d'Alessandro , M. R. Torrisi , A. Ferretti , M. C. Vulpiani , and V. Visco , “Extracorporeal Shock Wave Therapy Promotes Cell Proliferation and Collagen Synthesis of Primary Cultured Human Tenocytes,” Knee Surgery, Sports Traumatology, Arthroscopy: Official Journal of the ESSKA 19, no. 12 (2011): 2159–2168. 21617986 10.1007/s00167-011-1534-9

[29] P. Sripathi and D. K. Agrawal , “Rotator Cuff Injury: Pathogenesis, Biomechanics, and Repair,” Journal of Orthopaedics and Sports Medicine 6, no. 4 (2024): 231–248. 39574962 10.26502/josm.511500167 PMC11580759

[30] X. Feichtinger , X. Monforte , C. Keibl , et al., “Substantial Biomechanical Improvement by Extracorporeal Shockwave Therapy After Surgical Repair of Rodent Chronic Rotator Cuff Tears,” American Journal of Sports Medicine 47, no. 9 (2019): 2158–2166. 31206305 10.1177/0363546519854760

[31] C. J. Wang , H. Y. Huang , and C. H. Pai , “Shock Wave‐Enhanced Neovascularization at the Tendon‐Bone Junction: An Experiment in Dogs,” Journal of Foot and Ankle Surgery: Official Publication of the American College of Foot and Ankle Surgeons 41, no. 1 (2002): 16–22. 11858601 10.1016/s1067-2516(02)80005-9

[32] K. D. Kersh , S. R. McClure , D. Van Sickle , and R. B. Evans , “The Evaluation of Extracorporeal Shock Wave Therapy on Collagenase Induced Superficial Digital Flexor Tendonitis,” Veterinary and Comparative Orthopaedics and Traumatology: VCOT 19, no. 2 (2006): 99–105. 16810352

[33] Y. Chen , K. Lyu , J. Lu , et al., “Biological Response of Extracorporeal Shock Wave Therapy to Tendinopathy In Vivo (Review),” Frontiers in Veterinary Science 9 (2022): 851894. 35942112 10.3389/fvets.2022.851894 PMC9356378

[34] S. Huveneers and L. K. Phng , “Endothelial Cell Mechanics and Dynamics in Angiogenesis,” Current Opinion in Cell Biology 91 (2024): 102441. 39342870 10.1016/j.ceb.2024.102441

[35] Y. Zhou , D. Song , H. Liu , H. Li , B. Wang , and W. Sun , “Extracorporeal Shock Wave Therapy Alleviates Glucocorticoid‐Induced Injury and Dysfunction of Bone Microvascular Endothelial Cells via the PI3K/AKT/FOXO1 Pathway,” Annals of Joint 11 (2026): 5. 41657678 10.21037/aoj-25-36 PMC12875778

[36] Y. Z. Peng , K. Zheng , P. Yang , et al., “Shock Wave Treatment Enhances Endothelial Proliferation via Autocrine Vascular Endothelial Growth Factor,” Genetics and Molecular Research: GMR 14, no. 4 (2015): 19203–19210. 26782573 10.4238/2015.December.29.30

[37] K. Hatanaka , K. Ito , T. Shindo , et al., “Molecular Mechanisms of the Angiogenic Effects of Low‐Energy Shock Wave Therapy: Roles of Mechanotransduction,” American Journal of Physiology Cell Physiology 311, no. 3 (2016): C378–C385. 27413171 10.1152/ajpcell.00152.2016

[38] G. G. Schulze‐Tanzil , M. Delgado‐Calcares , R. Stange , B. Wildemann , and D. Docheva , “Tendon Healing: A Concise Review on Cellular and Molecular Mechanisms With a Particular Focus on the Achilles Tendon,” Bone & Joint Research 11, no. 8 (2022): 561–574. 35920195 10.1302/2046-3758.118.BJR-2021-0576.R1 PMC9396922

[39] Y. H. Chao , Y. H. Tsuang , J. S. Sun , et al., “Effects of Shock Waves on Tenocyte Proliferation and Extracellular Matrix Metabolism,” Ultrasound in Medicine & Biology 34, no. 5 (2008): 841–852. 18222032 10.1016/j.ultrasmedbio.2007.11.002

[40] L. Leone , M. Vetrano , D. Ranieri , et al., “Extracorporeal Shock Wave Treatment (ESWT) Improves In Vitro Functional Activities of Ruptured Human Tendon‐Derived Tenocytes,” PLoS One 7, no. 11 (2012): e49759. 23189160 10.1371/journal.pone.0049759 PMC3506633

