# Supplementary figures and images for: Effects of Extracorporeal Shock Wave Therapy on Tendon Integrity, Biomechanical Strength, Matrix Remodeling, Inflammation, Angiogenesis, and Tenogenic Differentiation in Rotator Cuff Injury
Source: Kaohsiung J Med Sci. 2026 Jul 14:e70260. Online ahead of print. doi: 10.1002/kjm2.70260 (PMC13399840; doi:10.1002/kjm2.70260)

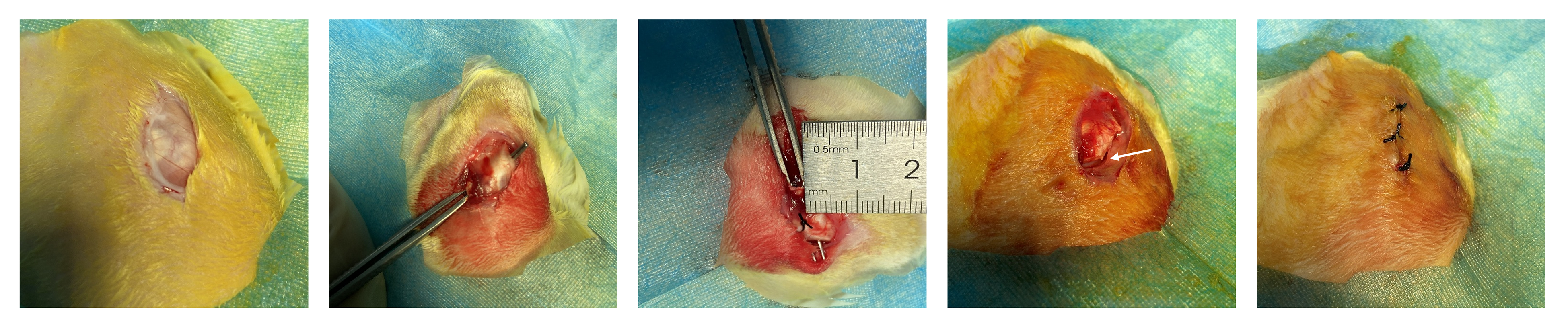

Supplement: Supplementary file 1 — Figure 1 Schematic diagram of RCI model establishment. [file KJM2-9999-e70260-s001.tif]
